# Nonlinear Dynamic Analysis of Pilotis Structures Supported by Drift-Hardening Concrete Columns

**DOI:** 10.3390/ma16196345

**Published:** 2023-09-22

**Authors:** Shiyu Yuan, Takashi Takeuchi, Yuping Sun

**Affiliations:** Department of Architecture, Graduate School of Engineering, Kobe University, Kobe 657-8501, Japan; yuanshiyu0510@gmail.com (S.Y.);

**Keywords:** pilotis story, concrete bearing-wall, nonlinear dynamic response, drift-hardening concrete column, ductile concrete column, residual drift, transient drift

## Abstract

Pilotis structures consisting of upper concrete bearing-walls and a soft first story have been well used in residential and office buildings in urban areas to primarily accommodate parking lots. In this research, drift-hardening concrete (DHC) columns developed by the authors are proposed to form the pilotis story with the aims of reducing its excessive residual drift caused by stronger earthquakes than anticipated in current seismic codes, mitigating damage degree, and enhancing resilience of the pilotis story. Nonlinear dynamic analysis was conducted to investigate the dynamic response characteristics of the wall structures supported by DHC columns. To this end, two sample six-story one-bay pilotis structures were designed following the current Japanese seismic design codes and analyzed. One sample structure is supported by ductile concrete (DC) columns, while the other is supported by DHC columns, which have the same dimensions, steel amount, and concrete strength as DC columns. Three representative ground motions were adopted for the nonlinear dynamic analysis. The analytical parameter was the amplitude of peak ground acceleration (*PGA*), scaled by the peak ground velocity (*PGV*) ranging between 12.5 cm/s and 100 cm/s with an interval of 12.5 cm/s. The analytical results have revealed that the residual drift of the pilotis story composed of DHC columns could be reduced to nearly zero under selected earthquakes scaled up to *PGV* = 100 cm/s, owing to not only the inherent self-centering ability of DHC columns but also the shake-down effect, which implies that the use of DHC columns can greatly enhance resilience of pilotis structures under strong earthquake inputs and promote its application in the buildings located in strong earthquake-prone regions. The maximum inter-story shear forces (MISFs) along the building height of the two models are also compared.

## 1. Introduction

Due to their high design flexibility and adaptability to the surrounding environment, pilotis structures, composed of upper bearing-walls and a soft first story, have been widely used in concrete buildings. Even in Japan, a country with high seismicity, to accommodate parking lots, the concrete pilotis structures have also found wide application in residential and office buildings constructed in urban areas with limited construction sites. While pilotis structures have many advantages from the viewpoint of aesthetics and function, the irregularity in lateral stiffness along building height is fatal to pilotis buildings located in earthquake-prone regions. Because the lateral stiffness of the soft first story is generally much lower than the upper bearing-walls, the lateral deformation induced by earthquakes tends to concentrate in the first story, cause severe damage to the columns there, and at worst make the building collapse, as analyzed by Mahin et al. [1].

To prevent concrete structures, inclusive of pilotis structures, from collapsing under strong design earthquakes, Japan extensively revised its Building Standards Law in 1981 and established a new seismic design methodology (NSDM), which is based on the concept of ultimate capacity design [2]. The NSDM consists of two levels of seismic design for building structures: the first level of seismic design aims at ensuring function and reparability of structural components under minor and/or moderate earthquakes, and the second level of seismic design is performed to verify if the structure can prevent buildings from major damages and/or collapse under design earthquake (DE) by allowing for ductile deformation and moderate damage in structural components. Since 1982, well-confined ductile concrete (DC) columns have been adopted in Japan to form the first story of pilotis buildings.

However, several post-1982 concrete pilotis structures were severely damaged and/or collapsed in the 1995 Hyogoken-Nanbu earthquake. According to the report by Building Research Institute (BRI) [3] on the damage survey of a total of 3938 concrete buildings located in the areas recorded with Japanese seismic intensity of 7.0 (the highest seismic scale adopted in Japan), the percentage of severely damaged and/or collapsed concrete pilotis structures was as high as 6.65% (=21/316), as compared with 2.4% for non-pilotis structures. It is noteworthy that two post-1982 concrete pilotis buildings collapsed, giving a percentage of 1.2%, which is much lower than the 5.37% for pre-1982 concrete pilotis buildings. Collapse and/or severe damage of concrete pilotis buildings conforming to modern seismic design codes were also observed in the 2008 Wenchuan earthquake and the 2016 Kumamoto earthquake [4]. Many researchers [5,6,7,8,9,10,11,12,13,14,15] have studied the collapse mechanism of concrete pilotis structures through numerical analysis and/or the shaking table test. Yoshimura [5] numerically analyzed the dynamic response of a post-1982 pilotis structure that had collapsed in the 1995 Hyogoken-Nanbu earthquake. The sample structure was supported by DC columns conforming to the NSDM. By taking degradation in the lateral resistance of DC columns into consideration, Yoshimura found that the collapse of the DC pilotis structure was mainly triggered by the excessive deformation of the soft first story combined with the axial load. A combination of excessive lateral deformation in the first story and the axial load results in a large P-Delta secondary moment that further degrades the lateral resistance of DC columns along with drift, leading to the collapse of the pilotis story.As described above, on one hand, seismic performance of concrete pilotis structures is generally inferior to that of non-pilotis structures. On the other hand, the pilotis structures have several important advantages over the non-pilotis structures. As summarized in references [3,16,17], most DC concrete pilotis structures conforming to modern design code provisions survived stronger earthquakes than DE anticipated in current codes. While large residual deformation was left in the first story of many DC pilotis structures, damage in the upper stories was very minor and reported in reference [15]. In other words, if the concrete columns forming the pilotis story are robust enough, the pilotis story can act like an isolator and significantly reduce damages in the upper living and acting stories. Furthermore, according to the report by the Tohoku Branch of AIJ [16] on the damage of buildings in the 2011 Eastern Japan earthquake, the low-story pilotis buildings located along coasts exhibited much higher tsunami-resisting performance than the non-pilotis ones.

To make the most of the above advantages and widen the application of pilotis structures, this paper proposes the use of drift-hardening concrete (DHC) columns in the first story. The DHC column is a new type of earthquake-resilient concrete component developed by the last author and his research team and can be easily made by just utilizing weakly bonded ultrahigh strength (referred to as SBPDN hereafter) rebars as the longitudinal reinforcement of concrete columns instead of deformed rebars. Sun et al. [18,19,20] have experimentally verified that the use of SBPDN rebars could ensure concrete columns’ sufficient drift-hardening capability up to the drift of 4.0% and more under high axial compression as the axial load ratio is 0.33. Other research studies [21,22] focus on increasing the post-yield stiffness by inserting unbonded rebars into an RC bridge pier, which still causes the yielding of longitude rebars and increases the complexity of fabrication.

**Figure 1:**
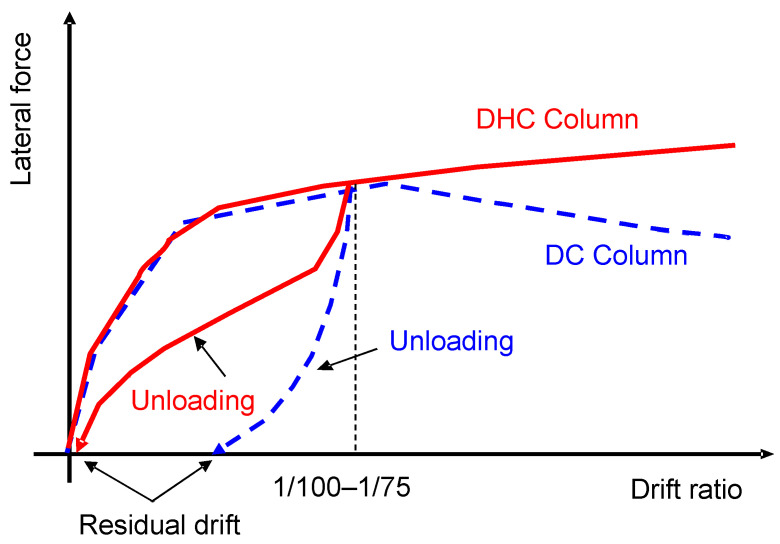
Comparison of mechanical properties of DHC and DC columns.

Figure 1 shows a comparison of the mechanical performances of DHC columns and conventional DC columns. As is obvious from Figure 1, DHC columns have two advantages over DC columns: (1) the lateral resistance of DHC columns stably increases along with drift ratio up to 4.0% and more, while that of DC columns tends to gradually degrade after the drift ratio of about 1.0–1.5%; (2) the residual drift of DHC columns can be kept as little as 1/10 of the transient drift up to at least 4.0% drift, but that of DC columns increases sharply after the transient drift exceeds 1.0–1.5%. Therefore, it can be presumed that the use of DHC columns in the first story will make pilotis concrete buildings resilient to both strong earthquakes and tsunamis.

To promote the application of the proposed DHC pilotis story, information on the dynamic response of the buildings supported by DHC columns is required. The objectives of this paper are (1) to investigate dynamic response characteristics of the pilotis structures supported by DHC columns through nonlinear dynamic analysis and (2) to verify the superiority of the proposed DHC pilotis structures to those supported by conventional DC columns, mainly from the perspective of the residual drift ratio in the pilotis story, which is the dominant index measuring damage degree.

## 2. Outline of the Sample Pilotis Buildings

**Figure 2:**
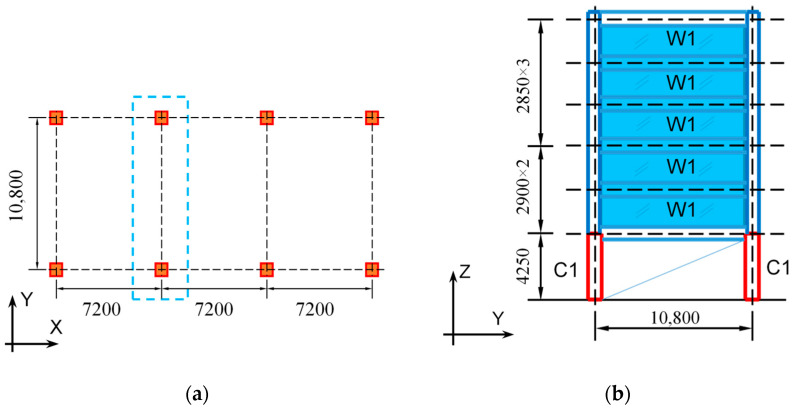
Outline of the sample pilotis building: (**a**) plan view; (**b**) elevation view. (Unit: mm.)

To achieve the goals of this study, two six-story one-bay concrete pilotis buildings were adopted and analyzed. The sample buildings represent typical pilotis residential apartments widely constructed during the 1980s to early 1990s in Japan and used in the previous analytical studies on the dynamic response of concrete pilotis structures supported by DC columns [6,7,8,9]. Figure 2 displays the plan view and elevation view in the Y direction of the sample buildings, which have identical geometrical dimensions. The X direction is a bare frame structure with a span of 7.2 m, while the Y direction is a bearing-wall structure from the second to sixth stories supported by the soft first story and has a span of 10.8 m. The pilotis structures in the Y direction will be analyzed. The unit weight of the second floor through the sixth floor was assumed to be 12.8 kN/m^2^, and that of the roof floor was 13.2 kN/m^2^ following the Japanese standard [23], which gave an axial load ratio of 0.14 to the columns in the pilotis story.

**Figure 3:**
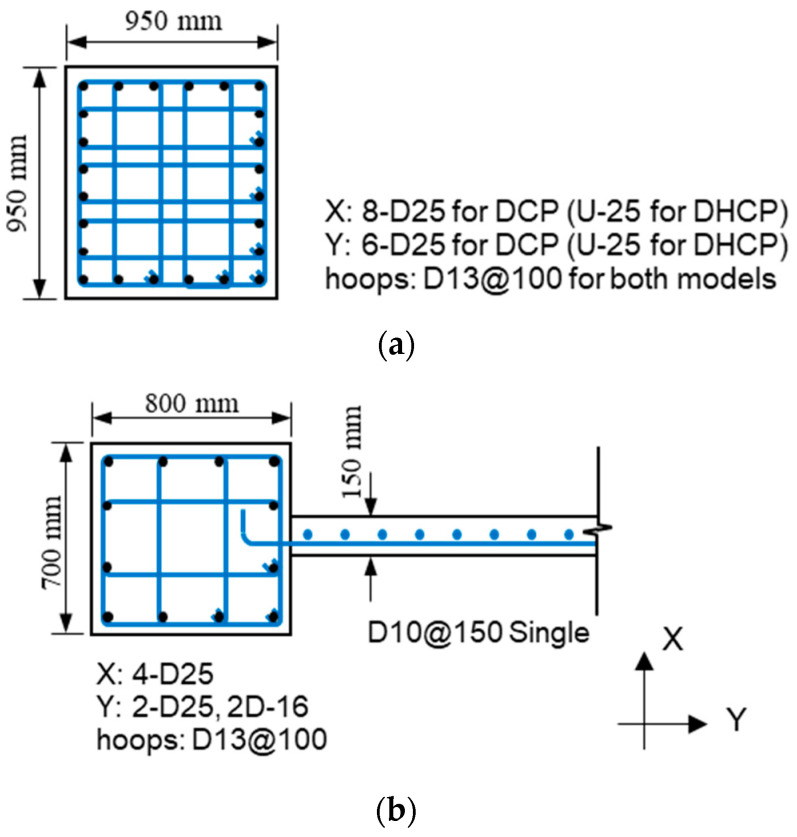
Sectional details of the structural elements: (**a**) column C1 at the first story; (**b**) wall W1 and the boundary column C2 at the upper stories.

The structural elements of two sample buildings have identical geometry, concrete strength, and reinforcement steel amounts. Sectional details of the column (C1) at the first story and the barbell-shaped wall at the upper stories are shown in Figure 3. The C1 column was a 950 mm square column reinforced by 24 rebars with a nominal diameter of 25 mm. The upper bearing wall (W1) consisted of a wall panel of 150 mm in thickness and two boundary rectangular columns (C2), whose dimensions were 700 mm in the X direction and 800 mm in the Y direction. The D10 deformed bars (295 MPa) were distributed in the wall panel, and the spacing of longitudinal and transverse reinforcement in the panel was 150 mm to give a steel ratio of 0.32%. The longitudinal rebars in the C2 columns consisted of eight D25 deformed bars and four D16 deformed bars, both of which have a specified yield strength of 345 MPa. D13 deformed bars (345 MPa) were used to confine both C1 and C2 columns with a spacing of 100 mm to give transverse steel ratios of 0.80% and 0.73%, respectively. The compressive strength of the concrete was 24 MPa.

The only difference between the two sample pilotis structures was the grade of longitudinal rebars in the C1 columns. One was normal-strength deformed bar (D-25 in Figure 3) having a specified yield strength of 345 MPa (SD345), and the other was ultrahigh strength bar (U-25 in Figure 3) with a specified yield strength of 1275 MPa (SBPDN). The SBPDN bar has a spiraled groove along its length and a much lower bond strength of about 3.0 MPa [24]. Hereafter, to distinguish the two pilotis structures, the one supported by C1 columns reinforced by SD345 rebars will be referred to as the DCP model and the other supported by C1 columns with SBPDN rebars as the DHCP model.

## 3. Method of Nonlinear Dynamic Analysis

**Figure 4:**
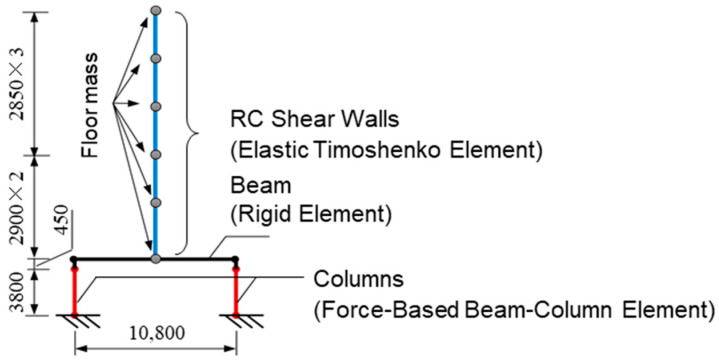
Two-dimensional frame model. (Unit: mm.)

**Figure 5:**
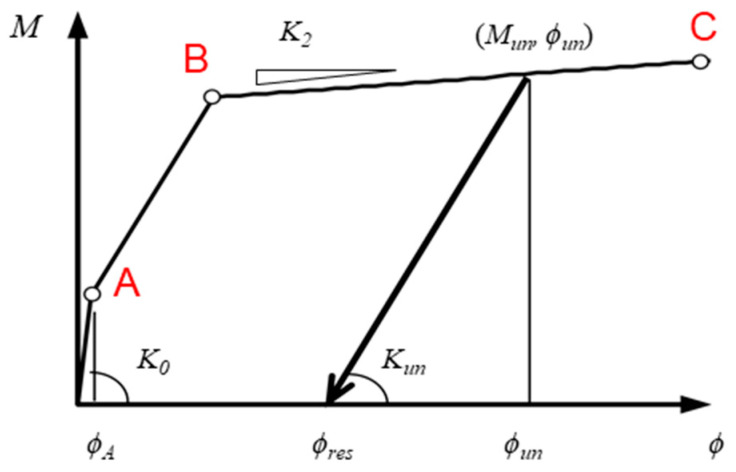
Hysteretic material model for the hinge zone moment-curvature relationship.

The Open system for earthquake engineering simulation (OpenSees, version 3.1.0) [25] was used to perform nonlinear dynamic response analysis for the two pilotis structures. The modeling of the pilotis structure is shown in Figure 4. The upper barbell-shaped shear walls were modeled as an elastic Timoshenko beam element [25] with the total mass of each floor lumped at the floor level, and the beam at the second-floor level was assumed to be a rigid beam. The bottom end of each column at the first story was assumed to be fixed to the foundation beam, and the top end had a rigid zone of 0.45 m to account for the effect of the rigid beam at the second-floor level. The columns at the pilotis story had a clear height of 3.8 m and were modeled as a force-based beam-column (FBBC) element [25]. Deformation of the FBBC element was calculated using the endpoint hinge integration (EHI) method proposed by Scott et al. [26]. The moment-curvature model of the column section in the hinge zone is the degrading-type tri-linear hysteretic material (HM) model [25], as shown in Figure 5. It is assumed that both DHC columns and DC columns have “perfect” ductility (no shear failure occurs) up to 4%. Although the ignorance of shear effects of the DC columns might lead to underestimation of the maximum and residual response [27], such disadvantages for DC columns were neglected to avoid complexity of the comparison.

Curvatures of the three points (A, B, and C) on the skeleton curve of the HM model correspond to the drift ratios of 0.25%, 1.0%, and 4.0%, respectively. Ignoring the elastic deformation of the column portion outside the hinge region, the relation of the curvature (*ϕ*) and the drift ratio (*R*) is expressed as:

(1) $$R=\phi \cdot L_{p}\left(1-L_{p}/2L\right),$$

where *L_p_* and *L* are the hinge length and the shear span of the column, respectively. The hinge length was assumed to be equal to the depth of the column section (900 mm), and the shear span was 1900 mm. The stiffness of unloading and reloading paths (*K_un_*) can be obtained from

(2) $$K_{un}=K_{o}\cdot \mu^{-\gamma },\;\mu =\phi_{un}/\phi_{A},$$

where *K_o_* is the initial stiffness, *ϕ_un_* and *ϕ_A_* are the curvature at unloading and reloading points and the curvature at point A (see Figure 5), respectively, and *γ* is the reduction factor of unloading and/or reloading stiffness.

To determine the ordinates (values of moment) of points A, B, and C on the skeleton curve, cyclical moment-curvature analysis of DHC and DC column sections was conducted using the method proposed by Sun et al. [24,28]. This method can account for the confinement effect by transverse reinforcement on concrete, the Bauschinger effect on cyclic unloading and reloading branches of longitudinal normal-strength (SD345) and ultrahigh strength (SBPDN) rebars, and the effect of slippage of SBPDN rebar on overall cyclic performance of the column section. Details of the constitutive laws of confined concrete and longitudinal reinforcement, along with the analytical procedures involved, can be found elsewhere [28] and will not be given here.

**Figure 6:**
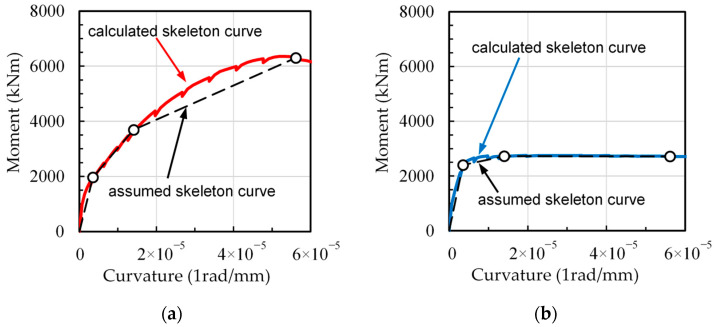
Modeling of the moment-curvature relationships for columns C1 at the first story: (**a**) DHC column; (**b**) DC column.

Figure 6 shows the calculated skeleton curves obtained by the analytical method [28] and the tri-linearized skeleton curves for the DHC column and DC column adopted in the two sample pilotis models. The calculated and assumed values of critical points on the tri-linearized skeleton models are listed in Table 1, where the parameter *α* represents the ratio of the third tangential stiffness of the skeleton model to the initial stiffness. The parameter *γ* has a large influence on the recovering degree of curvature after being unloaded from the transient curvature, and the larger the value of *γ* is, the less the residual curvature. The value of parameter *γ* = 0.5 shown in Table 1 for DHC columns was determined by best fitting the unloading and/or reloading straight lines in the moment-curvature model to the unloading and/or reloading curve of the calculated moment-curvature hysteresis. The value of 0.4 of *γ* shown in Table 1 for the DC columns has been widely used for nonlinear dynamic analysis of ductile concrete components [25].

## 4. Analytical Results and Discussions

### 4.1. Static Analysis

**Figure 7:**
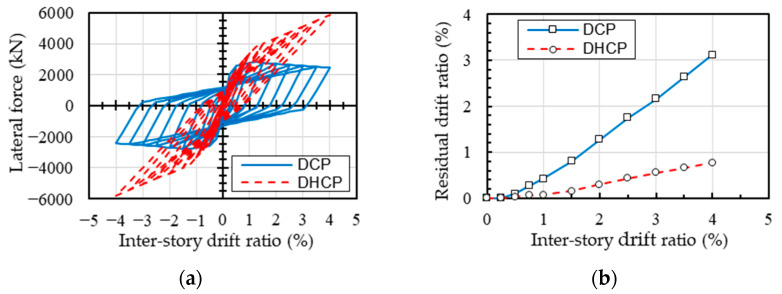
Comparison of static cyclic performance of the pilotis story of DHCP and DCP models: (**a**) the lateral force (*V*) versus inter-story drift ratio (*IDR*); (**b**) the residual drift ratio (*IDRres*) versus inter-story drift ratio (*IDR*) relationship.

Static nonlinear cyclic analysis was performed to better understand the fundamental difference between the DHCP and DCP models. The distribution of lateral force along the building height follows the Japanese building code provisions. This distribution has an inverse triangle shape. Because the upper bearing-walls remained in elastic stage, only the lateral force (*V*) versus inter-story drift ratio (*IDR*) relationship and the residual drift ratio (*IDRres*) versus *IDR* relationship of the pilotis story are compared and shown in Figure 7.

As one can see from Figure 7, the lateral resistance of the DHCP pilotis story stably increased along with deformation up to about 4.0% drift ratio, while that of the DCP pilotis story reached its peak at the drift ratio of 1.0% and then commenced gradually decreasing. The stable increase in lateral resistance of the DHCP pilotis story can be attributed to the low-bond strength of SBPDN rebars, which delays the yielding of longitudinal bars, enabling the lateral resistance by longitudinal rebars to increase along with deformation. The increase in lateral resistance by longitudinal rebars is large enough to cover the decrease caused by the inherent softening property of concrete and the *P*-Δ effect [19,20].

It is noteworthy that the residual drift ratio *IDRres* of the DHCP pilotis story was significantly smaller than that of the DCP pilotis story. At a transient drift ratio larger than 1.0%, the residual drift ratio of the former column was only about one-fourth of the latter one, implying that the DHCP pilotis story has a much superior recoverability to the conventional DCP pilotis story.

### 4.2. Dynamic Response Analysis

#### 4.2.1. Analysis Method and Input Ground Motions

The *Newmark-β* method was used to perform nonlinear dynamic analysis with *β* being 0.25. The Rayleigh damping was assumed for the pilotis models, and the damping ratio for the first and the second natural modes was assumed to be 0.03. The fundamental natural period of the DHCP model is 0.411 s, while that of the DCP model is 0.377 s. The former is a little larger than the latter because the initial stiffness of the DHCP model is lower than that of the DCP model (see Table 1).

**Figure 8:**
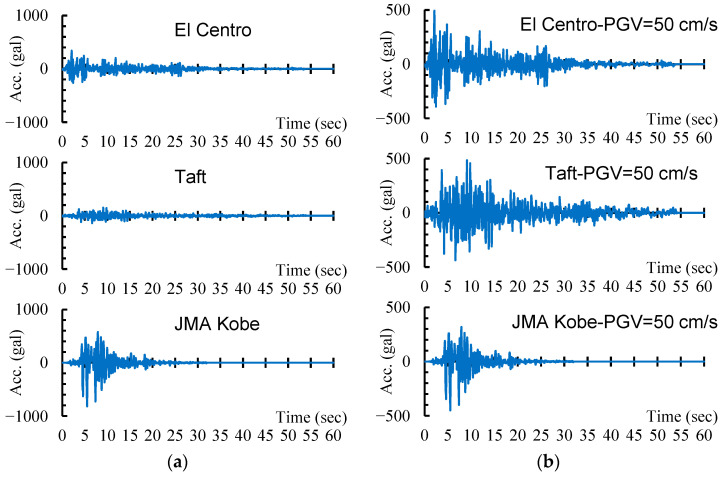
Ground acceleration histories: (**a**) original records; (**b**) scaled by *PGV* = 50 cm/s.

The NS components of three representative earthquake records are selected as the input ground motions for nonlinear incremental dynamic analysis (IDA). These ground motions are recorded in the 1940 El Centro earthquake, the 1952 Taft earthquake, and the 1995 Hyogoken-Nanbu earthquake, respectively. The first two earthquake records have been widely adopted in nonlinear dynamic analysis of building structures across the globe, and the third one, JMA Kobe, was recorded at Kobe Ocean Meteorological Observatory and had a maximum acceleration of 818 cm/s^2^ for the NS component. The peak ground acceleration (*PGA*) and the peak ground velocity (*PGV*) of the three selected earthquakes are listed in Table 2, along with the values of acceleration response *S_a_*(*T*_1_) and velocity response *S_v_*(*T_1_*), corresponding to the fundamental periods (*T*_1_) of the DHCP and DCP models with a damping ratio *h* = 0.05. Figure 8 displays the original ground acceleration histories and those scaled by *PGV* = 50 cm/s, the latter of which approximately corresponds to the safety limit of DE recommended in current Japanese design code. The selected earthquake records were scaled by the peak ground velocity (*PGV*) ranging from 12.5 cm/s to 100 cm/s with an interval of 12.5 cm/s in order to make sure the response maximum inter-story drift ratio of the pilotis story in the DHCP model was close to/beyond 4%.

#### 4.2.2. Maximum and Residual Inter-Story Drift Ratios

**Figure 9:**
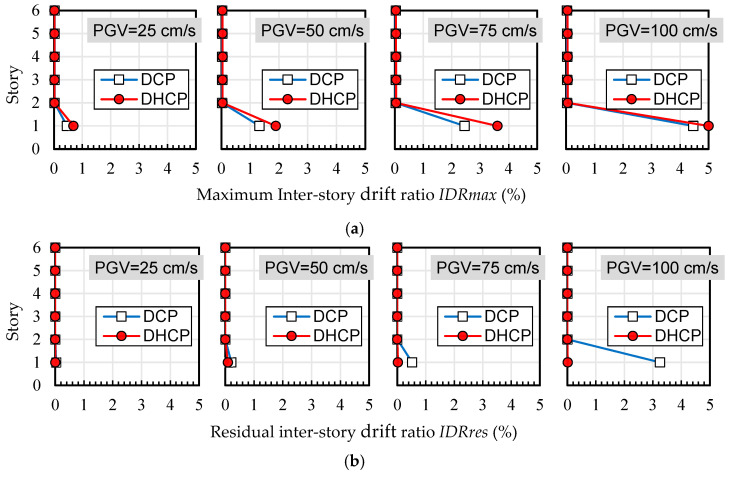
Maximum and residual inter-story drift ratios (El Centro): (**a**) maximum inter-story drift ratio *IDRmax*; (**b**) residual inter-story drift ratio *IDRres*.

**Figure 10:**
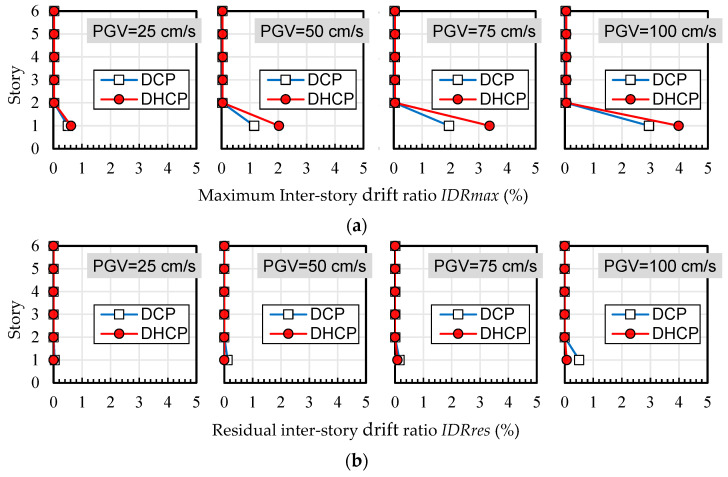
Maximum and residual inter-story drift ratios (Taft): (**a**) maximum inter-story drift ratio *IDRmax*; (**b**) residual inter-story drift ratio *IDRres*.

**Figure 11:**
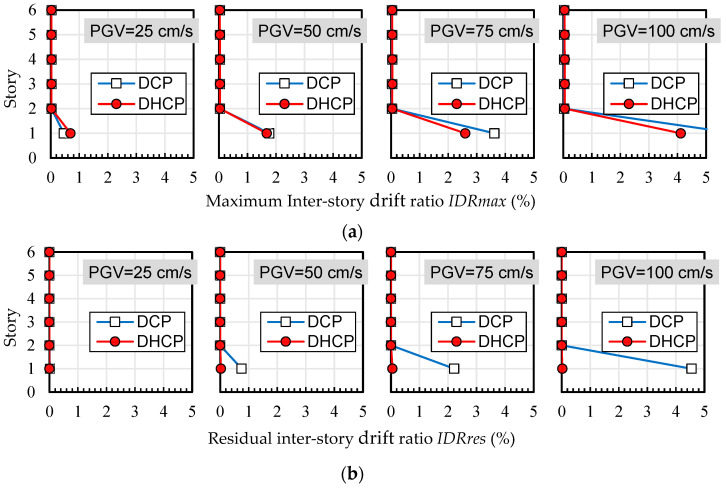
Maximum and residual inter-story drift ratios (JMA Kobe): (**a**) maximum inter-story drift ratio *IDRmax*; (**b**) residual inter-story drift ratio *IDRres*.

**Figure 12:**
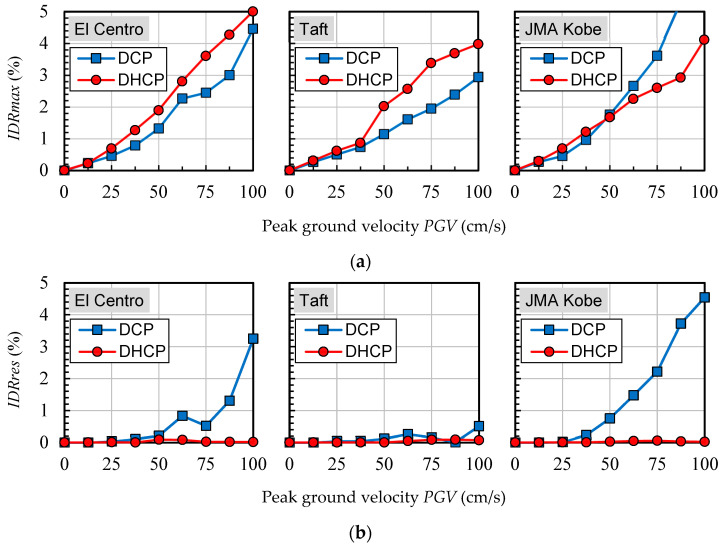
Maximum and residual inter-story drift ratios: (**a**) maximum inter-story drift ratio *IDRmax*; (**b**) residual inter-story drift ratio *IDRres*.

Maximum and residual inter-story drift ratios of each story level in the DHCP model and DCP model are compared in Figure 9, Figure 10 and Figure 11, corresponding to three selected earthquakes, respectively. Figure 12 shows the tendency of maximum and residual drift ratios along with the increase in *PGV* relationships in all of the input cases. In Figure 9, Figure 10 and Figure 11, four sets of analytical results of maximum and residual inter-story drift ratios are shown to correspond to four levels of earthquakes scaled by *PGV* = 25 cm/s (corresponds to damage limit of DE), 50 cm/s (corresponds to safety limit of DE), 75 cm/s, and 100 cm/s, respectively.

As is obvious from Figure 9, Figure 10 and Figure 11, the calculated maximum and residual drift ratios of the upper bearing-walls were very small, and lateral displacement was concentrated at the first story regardless of the differences in the earthquake records and their levels.

As shown in Figure 12, when subjected to the input cases of El Centro and Taft, maximum drift ratios at the first story of the DHCP model were 30–40% larger than those of the DCP model because the hysteretic energy dissipation capacity of DHC columns is poorer than DC columns, but the difference between the maximum drift ratios of the DHCP story and those of the DCP story did not diverge along with the earthquake intensity (the *PGV* value). It is worth noting from Figure 11a that under the input cases of JMA Kobe, the maximum drift ratio of the DHCP story became less than that of the DCP story when *PGV* was beyond 50 cm/s. The reason for this phenomenon will be discussed later.

One can see from Figure 9b, Figure 10b, Figure 11b and Figure 12b that the residual inter-story drift ratios of the DHCP story were kept close to zero even under extremely strong earthquakes scaled up to *PGV* = 100 cm/s for all three selected ground motions. This observation implies that a pilotis story composed of DHC columns is of very high recoverability after strong earthquakes. On the other hand, the residual drift ratios of the DCP story were very small when subjected to earthquakes scaled till *PGV* = 50 cm/s, approximately corresponding to the safety limit of DE prescribed in current Japanese seismic code, which indicates that the sample pilotis structures were properly designed. However, when subjected to stronger earthquakes than the safety limit of DE, in other words, when subjected to earthquakes scaled by *PGV* larger than 50 cm/s, the residual drift ratios of the DCP story commenced nonlinearly increasing along with *PGV*, particularly in the cases of the JMA Kobe and the El Centro earthquakes. In the case of the Taft earthquake, the residual drift ratios of the DCP story subjected to strong earthquakes with *PGV* larger than 50 cm/s were much less than those under the JMA Kobe and the El Centro earthquakes.

**Figure 13:**
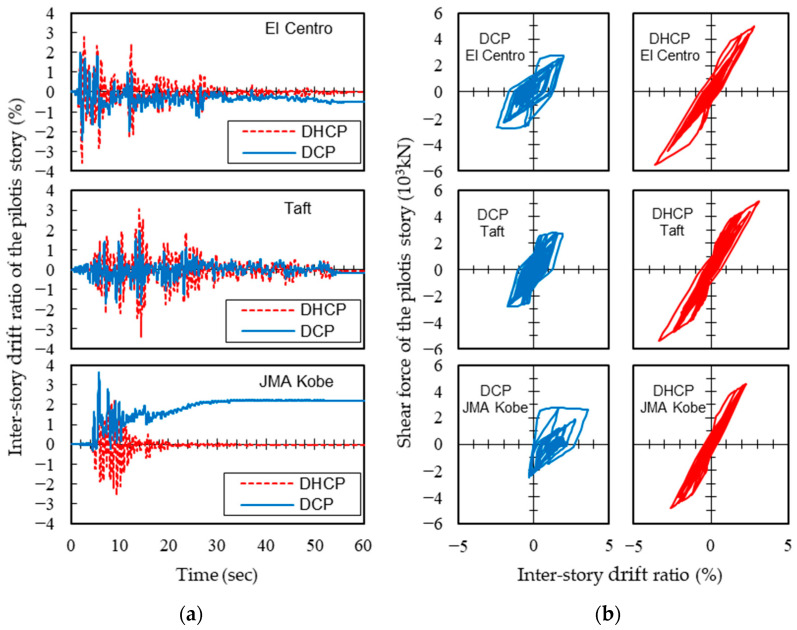
Response histories of inter-story drift ratios and inter-story shear force at pilotis story (*PGV* = 75 cm/s): (**a**) time histories of inter-story drift ratio *IDR*; (**b**) inter-story shear force versus inter-story drift ratio *ISF*-*IDR*.

To better understand why the maximum drift ratios of the DCP story became larger than those of the DHCP story when subjected to the JMA Kobe earthquake input with *PGV* larger than 50 cm/s (as shown in Figure 12), the time histories of inter-story drift ratios of the DHCP and DCP pilotis stories along with their story shear force versus drift ratio (*ISF*-*IDR*) responses under the JMA Kobe earthquake scaled by *PGV* = 75 cm/s are compared in Figure 13. For comparison, the analytical results under the El Centro and the Taft earthquakes are also displayed in Figure 13.

As is obvious from Figure 13, the DHCP story vibrated reversely around the elevational axis of the building with nearly zero residual drift ratio under all three ground motions, and the *ISF*-*IDR* responses exhibited very clear self-centering characteristics. The DCP story also reversely vibrated around the axis of the building when subjected to the El Centro and the Taft ground motions, but when subjected to the JMA Kobe earthquake, from time *t* = 10 s onward, response deformation of the DCP story commenced shifting to the plus side. After 15 s, the residual drift ratio further began to gradually accumulate along with the time and reached about 2.2% at the end of ground motion. This can be attributed to the fact that the JMA Kobe earthquake contains typical pulse-like ground motion, which displaced the DC columns greatly beyond their yielding displacement and hindered the reverse vibration of the columns.

**Figure 14:**
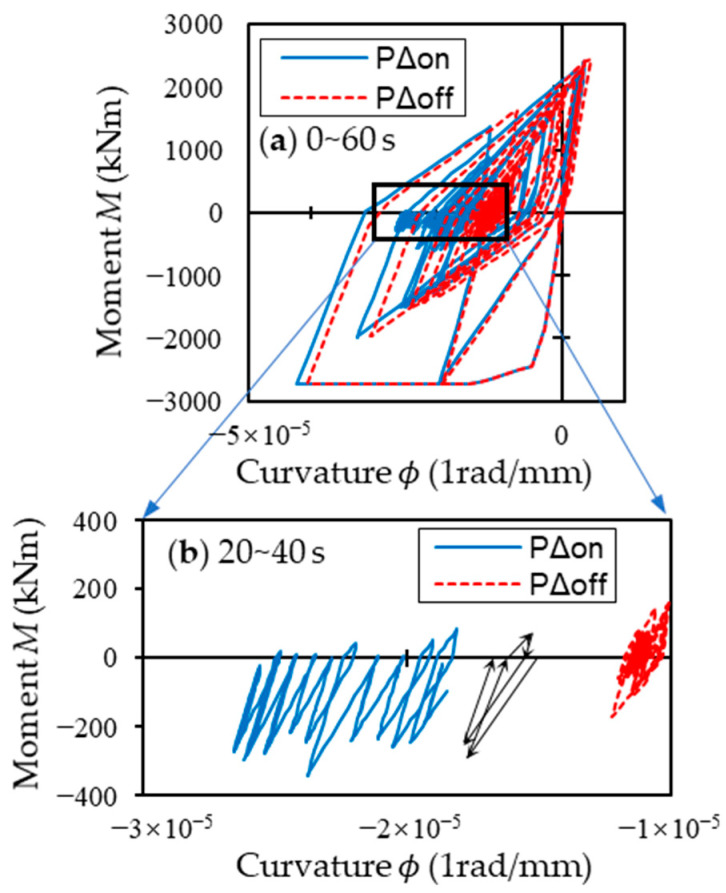
The moment-curvature response histories at the left column C1 bottom hinge sections of DCP model considered or ignored the *P*-Δ effect: (**a**) time histories of full dynamic response time 0–60 s; (**b**) extracted from 20 to 40 s, the black arrow lines demonstrate the accumulation of drift ratio of DC model in 1.5 cycles of the response history when *P*-Δ effect was considered.

To investigate what caused the one-sided gradual accumulation of inter-story drift ratio of the DC story after t = 15 s, influence of the so-called *P*-Δ effect is shown in Figure 14, which displays moment-curvature responses of the C1 column at the DCP story with the *P*-Δ effect being considered and/or not when subjected to the JMA Kobe earthquake scaled by *PGV* = 75 cm/s. As is apparent from Figure 14, it is the *P*-Δ effect that caused the accumulation of drift ratio of the DC story little-by-little along with vibration time. After the pulse-like input of motion caused large plastic deformation in the DC columns, the moment varied only on one side because of the effect of the *P*-Δ moment, resulting in the gradual accumulation of drift ratio along the time.

#### 4.2.3. Influence of Dynamic Loading on Residual Drift Ratio

**Figure 15:**
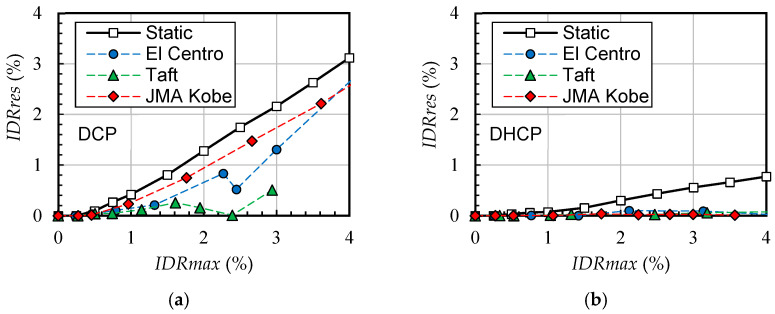
Residual drift ratio versus maximum drift ratio relationships: (**a**) DCP model; (**b**) DHCP model.

By comparing Figure 7 and Figure 12, one can see that the residual inter-story drift ratios of the sample DHCP story subjected to earthquakes (random dynamic loadings) were much smaller than those subjected to static cyclic loading. To investigate the influence of dynamic loading on the residual drift ratios, Figure 15 displays residual drift ratio versus maximum drift ratio relationships for the two sample pilotis models. In Figure 15, the solid line with white square marks represents the results obtained under static cyclic loading, and the other three dash lines with blue circle marks, green triangle marks, and red diamond marks express those under the input cases of El Centro, Taft, and JMA Kobe, respectively.

As can be seen from Figure 15, for the conventional DCP pilotis story, the dynamic loading induced by earthquakes could reduce the residual inter-story drift ratio, but the reduction degree varies within three selected earthquake records. The Taft input cases most significantly reduced the residual drift ratios of the DC story corresponding to the maximum drift ratio of 3.0% below the threshold drift ratio of 0.5%, which represents the residual drift ratio defined in the FEMA P-58 [29] for damage state 2 (DS2) of concrete structures, while the static residual drift ratio was as high as 2.2% after being unloaded from the 3.0% drift ratio. However, the reduction in residual drift ratio by dynamic loading was very small in the case of JMA Kobe.

For the DHCP pilotis story, relatively, the effect of dynamic loading on the reduction in residual drift ratio was very significant under all the selected earthquake records. Due to the large drift-hardening capability and significant self-centering ability of DHC columns [18,19,20], the residual drift ratio of the DHCP story was already much less than that of the DCP story under static cyclic loading as shown in Figure 7 and Figure 12; the residual drift ratio of the DHCP story was further reduced close to zero even when subjected to extremely strong earthquakes scaled up to *PGV* = 100 cm/s along with a maximum inter-story drift ratio around 4%. Furthermore, the effect of dynamic loading (earthquakes) on reducing the residual drift ratio of DHCP was not affected by the maximum drift ratio under three selected earthquake records.

**Figure 16:**
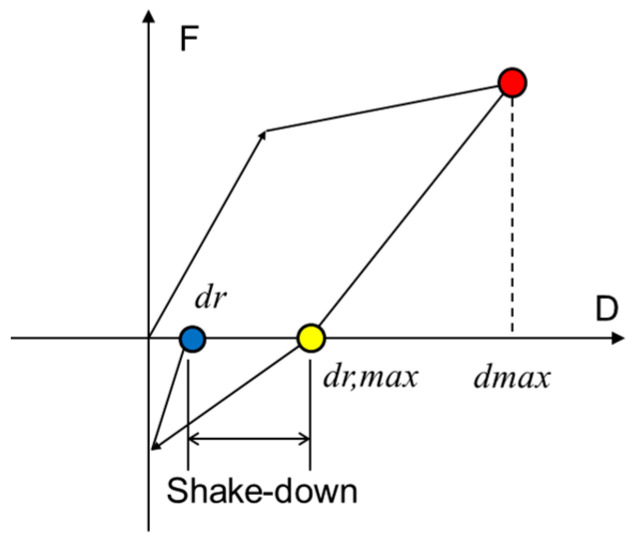
Concept of shake-down effect.

The further reduction in residual drift ratio for DCP and DHCP stories can be attributed to the so-called shake-down effect. As pointed out by MacRae and Kawashima [30] and shown in Figure 16, the residual drift *dr* after dynamic response is not only affected by the static mechanical property of the structural members but also influenced by the vibrating history and tends to become less than the static residual drift ratio *dr,max*.

#### 4.2.4. Maximum Inter-Story Shear Force

**Figure 17:**
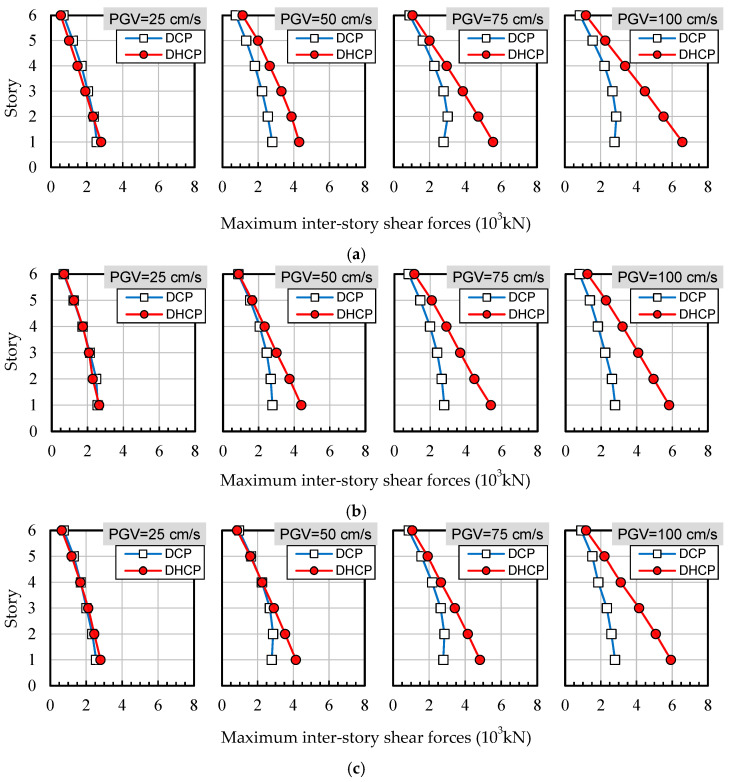
Maximum inter-story shear forces (MISFs) along the building height for the three selected earthquake records and at four levels of ground motions scaled by *PGV* = 25 cm/s, 50 cm/s, 75 cm/s, and 100 cm/s: (**a**) El Centro; (**b**) Taft; (**c**) JMA Kobe.

To see the difference in the performance of the upper bearing-walls in the sample DHCP and DCP models, maximum inter-story shear forces (MISFs) along the building height are compared in Figure 17 for the three selected earthquake records and at four levels of ground motions scaled by *PGV* = 25 cm/s, 50 cm/s, 75 cm/s, and 100 cm/s, respectively.

As shown in Figure 17, when subjected to the earthquakes scaled by *PGV* = 25 cm/s, MISFs of the upper bearing-walls in the DHCP and DCP models exhibited nearly identical distribution along the building height and had almost the same amplitude regardless of the difference among the earthquake records. As the *PGV* increased, the MISFs in the DHCP model tended to become larger than those in the DCP model. When subjected to earthquakes with *PGV* = 50 cm/s and larger, the distribution of the MISFs along the building height of the DHCP model began to differ from that of the DCP model.

It is particularly worth noting that the MISFs for the upper bearing-wall in the DCP model exhibited nonlinear distribution along the building height and that the inter-story shear force at the bearing-wall stories adjacent to the pilotis story in the DCP model did not increase along with the increase in *PGV* value but converged to a value that is close to the ultimate lateral resistance (see Figure 7a) by the DC columns forming the first story. On the other hand, the inter-story shear force at the bearing-wall adjacent to the pilotis story in the DHCP model did increase along with *PGV* value, and the distribution of the MISFs of the upper bearing-wall in the DHCP model can be approximately linearized. The increase in the inter-story shear force at the bearing-wall adjacent to the pilotis story in the DHCP model can be attributed to the high drift-hardening capability of the DHC columns forming the pilotis story. As shown in Figure 17, the maximum inter-story shear force is nearly equal to the sum of lateral resistances of the two DHC columns.

Two observations can be made from Figure 17 about the DHCP buildings. The first is that the upper bearing wall should be designed to have sufficiently large lateral resistance to make full use of the large drift-hardening capability and high recoverability of DHC columns. The second is that if the upper bearing-wall does not have ultimate lateral resistance large enough to exceed that of the DHC columns at the pilotis story, the pilotis story will not be the weak point in a pilotis-type building, which implies that the use of DHC columns could make a pilotis story robust enough to make pilotis buildings resilient to both strong earthquakes and tsunamis.

One should be aware that certain response values of a pilotis building supported by DHC columns such as *IDRmax*, *IDRres,* and *MISFs* could be affected by the selection of earthquake records [31,32]. To evaluate maximum drift demands of such buildings within a certain region, soil type, target design spectrum, etc., and, furthermore, to propose a design method for such buildings requires the appropriate selection of records.

## 5. Conclusions

To develop concrete pilotis structures resilient to stronger earthquakes than anticipated in modern seismic design codes and tsunamis, the use of drift-hardening concrete columns to form the first story was proposed. Nonlinear incremental dynamic analysis was performed to investigate dynamic response characteristics of the pilotis structures supported by the DHC columns. Three representative earthquake records were selected for the dynamic analysis. Through analytical results and discussions described in this paper, the following conclusions can be drawn.

1. The use of DHC columns to support the upper bearing-wall could ensure pilotis buildings sufficient robustness and significantly enhance their resilience. Even subjected to extremely strong earthquakes scaled up by *PGV* = 100 cm/s, the residual drift ratio of the DHCP story remained close to zero, implying the high recoverability and re-occupancy of the DHCP buildings;
2. Not only the inherent self-centering ability but also the shake-down effect contributed to the reduction in residual drift ratio in the DHCP story. On the other hand, the reduction in drift ratio in the DCP story by the shake-down effect varies with earthquake records and should not be expected for the pilotis buildings supported by conventional DC columns;
3. To make the most of the high resilience of the DHCP story, the upper bearing-wall should have a larger lateral resistance than the sum of the DHC columns. In other words, if the upper bearing-wall does not have sufficient ultimate lateral resistance, the DHCP story will no longer be the weak point of a pilotis building; careful structural design of the upper bearing-wall is needed to ensure high recoverability of the whole building;
4. Because the hysteresis energy dissipation capacity of the DHC columns was poorer than that of the DC columns, the DHCP story generally exhibited larger lateral deformation (larger maximum inter-story drift response) than the DCP story. When subjected to the JMA Kobe earthquake, which is a pulse-like earthquake, however, the maximum inter-story drift ratio of the DHCP story was smaller than that of the DCP story as *PGV* became larger than 50 cm/s.

Further investigation is required to evaluate the maximum drift demands of pilotis buildings supported by DHC columns corresponding to building regions, soil types, target design spectrums, etc.

## Figures and Tables

**Table 1:** Parameters defining the moment-curvature models of DHC and DC columns.

Column Model	*M_A_* (kN∙m)	*ϕ_A_* (rad/mm)	*M_B_* (kN∙m)	*ϕ_B_* (rad/mm)	*M_C_* (kN∙m)	*ϕ_C_* (rad/mm)	*α*	*γ*
DHC	1970	0.33 × 10^−5^	3690	1.40 × 10^−5^	6410	5.60 × 10^−5^	0.110	0.5
DC	2400		2730		2720		−0.002	0.4

**Table 2:** Primary parameters of the selected three earthquake records.

Record	*PGA* (cm/s^2^)	*PGV* (cm/s)	*S_a_* (*T*_1_) (cm/s^2^)		*S_v_* (*T*_1_) (cm/s)	
			DHCP	DCP	DHCP	DCP
El Centro ^1^	341.7	33.5	577.3	665.0	37.8	39.9
Taft ^1^	152.7	15.7	422.4	353.9	25.3	23.1
JMA Kobe ^2^	818.0	90.7	2128.2	2406.9	139.2	144.4

^1^ Provided by The Building Center of Japan (BCJ). ^2^ Provided by Japan Meteorological Agency (JMA).

## Data Availability

The data presented in this study are available on request from the corresponding author.
